# Postoperative anaemia: the unseen challenge in cardiac surgery

**DOI:** 10.1016/j.bjao.2025.100514

**Published:** 2025-12-17

**Authors:** Matthew A. Warner, Jacob Raphael

**Affiliations:** 1 Department of Anesthesiology and Perioperative Medicine, Mayo Clinic, Rochester, MN, USA; 2 Department of Anesthesiology, Thomas Jefferson University, Philadelphia, PA, USA

**Keywords:** anaemia, cardiac surgery, haemoglobin, iron deficiency, patient-centred outcomes, patient blood management, postoperative, transfusion

## Abstract

Postoperative anaemia is an overlooked complication of cardiac surgery that is associated with adverse clinical outcomes. Although small clinical trials suggest that postoperative treatment with i.v. iron improves haemoglobin recovery and reduces transfusion utilisation, appropriately powered randomised controlled trials are necessary to definitively evaluate the efficacy of treatment on clinical outcomes of importance to patients, clinicians, and healthcare systems. A comprehensive approach to perioperative anaemia management demands a renewed focus on both prevention and treatment to improve patient outcomes.

Preoperative anaemia is a modifiable risk factor for adverse cardiac surgical outcomes.^1^, ^2^, ^3^, ^4^ Clinical trials demonstrate that treatment of anaemia in advance of surgery improves haemoglobin recovery and reduces allogeneic transfusions,^5^, ^6^, ^7^ with a large forthcoming trial evaluating the impact on patient-centred outcomes.^8^ Preoperative anaemia treatment has been endorsed in multiple clinical practice guidelines.^9^, ^10^, ^11^, ^12^ Yet, only a small percentage of patients with anaemia before cardiac surgery receives dedicated anaemia evaluations, and an even smaller proportion receives anaemia-directed therapies.^13^^,^^14^ Hence, although many consider treatment of preoperative anaemia a standard of care,^15^ the reality is that implementation remains sparse.

In this issue, Alkadri and colleagues^16^ focus on a related but distinct clinical entity—postoperative anaemia occurring after cardiac surgery. Despite the near ubiquity of anaemia after cardiac surgery, few studies have evaluated its association with clinical outcomes. Specifically, the authors evaluated the association between the nadir haemoglobin concentration on postoperative day 1 and a primary composite outcome of 30-day mortality, stroke, myocardial infarction, acute kidney injury, and deep sternal wound infection. After analysing a cohort of 5960 patients, they report that each 10 g L^–1^ decrease in haemoglobin was associated with a 1.15 (95% confidence interval [CI] 1.05–1.25) increase in the odds of the primary outcome after adjustment for key covariates, including preoperative haemoglobin, perioperative transfusions, and surgical features. Further, a nadir haemoglobin <80 g L^–1^ was associated with a 1.44 (95% CI 1.19–1.75) increase in the odds of the primary composite outcome.

By using continuous haemoglobin concentrations as their primary exposure rather than arbitrary bins of anaemia severity, the authors were able to identify an inflection point for increasing risk as the haemoglobin concentration decreases below 10–11 g dl^–1^. This is consistent with data from several other surgical populations,^17^, ^18^, ^19^ providing a potential target at which treatment should be evaluated in clinical trials. There were no significant interactions by patient sex, highlighting that postoperative anaemia is similarly accompanied by adverse clinical outcomes in both women and men. Strengths of the study include predefined exposures and outcomes with mechanistic plausibility, careful adjustment for confounding variables, and the use of several predefined modelling approaches to ensure consistency of study findings. Limitations primarily stem from the retrospective study design; thus, these observational data represent associations and do not establish causality, nor do they present treatment options or the potential for treatment modification. Further, patient-centred recovery metrics that have mechanistic links to tissue-mediated oxygen delivery, such as fatigue, physical function, cognition, and quality of life, were not evaluated.

Given the associations between postoperative anaemia and adverse clinical outcomes, should we treat patients with postoperative anaemia and how should we treat them? Several small trials have evaluated administration of iron after cardiac surgery to address postoperative anaemia. Iron is the clear frontrunner for postoperative anaemia treatment. Iron is essential for erythrocyte production, and iron availability may be limited by bleeding-related iron losses, preexisting iron deficits, or perioperative iron sequestration secondary to inflammation.^20^ I.V. iron offers several advantages over p.o. iron, including faster iron replenishment and higher compliance with therapy*.* Kremke and colleagues^21^ compared a single 1000 mg dose of i.v. iron *vs* daily p.o. iron for 100 adults after cardiac surgery. Those receiving i.v. iron were less likely to remain anaemic 4 weeks later and had lower transfusion utilisation from day 4 through 40 after surgery. In another small trial of 100 critically ill adults, approximately half post-cardiac surgery, anaemia treatment with i.v. iron compared with standard care was accompanied by greater haemoglobin recovery, numerically lower transfusion utilisation, and with effect estimates suggesting potential positive effects on fatigue, physical function, cognition, and mental health through 3 months after hospitalisation.^22^ Despite these promising data, appropriately powered RCTs are necessary to definitively evaluate the efficacy of i.v. iron on clinical outcomes of importance to patients, clinicians, and healthcare systems. Fortunately, i.v. iron is broadly accessible and has an established safety profile, with self-limited infusion reactions occurring in <5% of patients.^23^ Furthermore, postoperative treatment can improve access to care given that all postoperative cardiac surgery patients remain hospitalised for several days after surgery. Yet, the costs of therapy, which vary broadly based on formulation, dose, and geographic location, may preclude cost-effectiveness in the absence of demonstrable clinical benefit.

Should we abandon preoperative anaemia management in favour of anaemia treatment after surgery? The challenges of preoperative anaemia management, primarily establishing the requisite clinical space, staffing, infrastructure, and pathways to evaluate and treat patients with modalities such as i.v. iron in an expedient fashion before surgery, are largely overcome in the postoperative setting. I.V. iron may be readily administered during inpatient admissions given that trained personnel and equipment are already in place. An ongoing pilot trial is evaluating postoperative treatment with i.v. iron for patients identified as having iron deficiency anaemia before surgery, thereby shifting treatment from the preoperative to postoperative period.^24^ Although treatment after surgery is likely warranted for those with established iron deficiency who are unable to be treated before surgery because of time constraints or lack of treatment access, we should avoid the temptation to forgo preoperative anaemia management altogether. By addressing anaemia before surgery, not only do we improve haematologic readiness for the surgical insult but also attenuate the severity of anaemia after surgery, exemplifying the adage that ‘an ounce of prevention is worth a pound of cure’. Further, preoperative treatment with i.v. iron results in sustained haemoglobin recovery for many months after surgery.^25^ There are examples of success in preoperative anaemia implementation in cardiac surgery,^26^^,^^27^ and such programmes improve not only clinical outcomes but also hospital finances.^28^ Patients with established anaemia may benefit from combined treatment both before and after surgery; however, future trials are necessary to define optimal treatment approaches for those with incident postoperative anaemia and progression of preexisting anaemia.

Beyond treatment, a comprehensive approach also demands a renewed focus on prevention. After ensuring adequate preoperative optimisation of red cell mass and iron stores, we must use intraoperative blood conservation strategies to reduce the quantity of surgical blood loss. This includes the use of minimally invasive surgical approaches when possible and meticulous surgical technique, antifibrinolytics, optimisation of cardiopulmonary bypass circuits (e.g. modification of circuit size, minimisation of prime volume, retrograde autologous priming), use of cell salvage, and the use of point-of-care coagulation testing and treatment algorithms to guide haemostatic therapies. After surgery, it is essential to closely monitor for bleeding with prompt correction of coagulopathy or surgical exploration in the absence of coagulopathy and the avoidance of further iatrogenic blood loss through the optimisation of phlebotomy practices (e.g. minimisation of laboratory draws, use of low-volume blood collection tubes). This holistic approach to anaemia prevention and treatment throughout the entire spectrum of patient cares falls within the patient blood management framework, which has been recognised by multiple societies and the World Health Organization as a key priority for improving patient outcomes.^29^, ^30^, ^31^

In summary, the study by Alkadri and colleagues^16^ is a welcome addition to the literature that highlights postoperative anaemia as an overlooked complication of surgery that is associated with adverse clinical outcomes. Having solidified the groundwork for the potential risks of postoperative anaemia, it is now time for large clinical trials to evaluate postoperative anaemia treatment strategies, focusing on outcomes of greatest importance to patients as they recover from cardiac surgery.

## Declarations of interest

MAW received an honorarium from Pharmacosmos for a presentation at World Anemia Day 2025 and receives research support from the National Heart, Lung, and Blood Institute (NHLBI) of the National Institutes of Health (NIH), outside of the submitted work. The contents of this manuscript are solely the responsibility of the authors and do not necessarily represent the official views of the NIH.

JR reported receiving research support and honorarium from CSL Behring and receiving honorarium from Octapharma outside of the submitted work.
